# Monte Carlo simulation of secondary neutron dose for scanning proton therapy using FLUKA

**DOI:** 10.1371/journal.pone.0186544

**Published:** 2017-10-18

**Authors:** Chaeyeong Lee, Sangmin Lee, Seung-Jae Lee, Hankyeol Song, Dae-Hyun Kim, Sungkoo Cho, Kwanghyun Jo, Youngyih Han, Yong Hyun Chung, Jin Sung Kim

**Affiliations:** 1 Department of Radiological Science, Yonsei University, Wonju, Korea; 2 Program in Biomedical Radiation Sciences, Department of Transdisciplinary Studies, Graduate School of Convergence Science and Technology, Seoul National University, Seoul, Republic of Korea; 3 Department of Radiation Oncology, Samsung Medical Center, Sungkyunkwan University School of Medicine, Seoul, Republic of Korea; 4 Department of Radiation Oncology, Yonsei Cancer Center, Yonsei University College of Medicine Seoul, Seoul, Republic of Korea; North Shore Long Island Jewish Health System, UNITED STATES

## Abstract

Proton therapy is a rapidly progressing field for cancer treatment. Globally, many proton therapy facilities are being commissioned or under construction. Secondary neutrons are an important issue during the commissioning process of a proton therapy facility. The purpose of this study is to model and validate scanning nozzles of proton therapy at Samsung Medical Center (SMC) by Monte Carlo simulation for beam commissioning. After the commissioning, a secondary neutron ambient dose from proton scanning nozzle (Gantry 1) was simulated and measured. This simulation was performed to evaluate beam properties such as percent depth dose curve, Bragg peak, and distal fall-off, so that they could be verified with measured data. Using the validated beam nozzle, the secondary neutron ambient dose was simulated and then compared with the measured ambient dose from Gantry 1. We calculated secondary neutron dose at several different points. We demonstrated the validity modeling a proton scanning nozzle system to evaluate various parameters using FLUKA. The measured secondary neutron ambient dose showed a similar tendency with the simulation result. This work will increase the knowledge necessary for the development of radiation safety technology in medical particle accelerators.

## 1. Introduction

Proton therapy is a rapidly expanding field of cancer treatment [1, 2]. The protons have potential advantages of delivering higher dose to the cancer and lower dose to the normal surrounding tissue. This highly precise localization is achieved by the Bragg peak effect, which allows a sharp distal fall-off depth dose distribution [3]. For the above reasons, proton therapy facilities are being construction globally, and there will be more than 100 proton therapy centers globally within a few years.

Although proton therapy itself has outstanding physical advantages over X-ray treatment, there are some problems and limitations [4–5]. One of the main problems is the production of secondary neutrons when the protons undergo nuclear interactions with patients or components inside the nozzle [5]. The secondary neutrons that are generated from protons have a higher Relative Biological Effectiveness than photons [6–7]. Therefore, side effects to the patient may occur. For instance, the secondary neutron is one of the factors that increase the integral dose, which can lead to the patient’s secondary cancer [8].

There have been a variety of studies related to the induced secondary neutrons during proton therapy [9–12]. Proton therapy centers have reported simulated and experimental results regarding induced secondary neutrons. It is difficult to directly compare the results from different centers because each treatment center has their own proton facility design and beam conditions. Because the nozzle design would be changed according to the purpose even in the same scanning nozzle, it is necessary to consider the neutron dose that is caused by the differences between each proton nozzle.

In this study, the production of secondary neutrons was evaluated for two different gantries in the same scanning mode. First of all, proton therapy scanning nozzles were modeled by using FLUKA Monte Carlo code [13], and then we performed the analysis of the induced secondary neutrons from Gantry 1. After beam properties of each nozzle were validated, simulations and experiments were performed to evaluate the induced secondary neutrons from the nozzles according to detector position.

## 2. Materials and methods

### 2.1 Validation of proton beam properties

In the Samsung Medical Center (SMC), there are two fully rotating gantries with different nozzles, which utilize protons that can be accelerated up to 230 MeV using a conventional cyclotron. Table 1 shows properties of the different nozzles. Gantry 1 can be operated in wobbling and scanning modes, but Gantry 2 employs the scanning mode only. Gantry 1 can operate in a similar mode to the scanning mode of Gantry 2, but does not contain a pipe through which He gas pipe can be added [14]. The helium gas pipe reduces the proton beam penumbra.

**Table 1:** Explanation of the different proton nozzles at SMC.

Nozzle	Type	Explanation / Principle
Gantry 1: Multipurpose nozzle	Wobbling mode	- For wobbling mode operation, the two *x* and *y* wobbling magnets will widen the proton beam, which will then pass through the scatterer to form a two-dimensional dose distribution, which is the actual field size. - To deliver the correct depth of dose to the tumor, the ridge filter forms a spread-out Bragg peak by alternating the pristine Bragg peak.
Gantry 2: Pencil Beam Scanning dedicated nozzle	Scanning mode	- Several components used for the wobbling mode are not required, such as the wobbling magnets, scatterers, ridge filters, MLC, and compensator.
		- In the PBS dedicated nozzle, there are far fewer components, which include a scanning magnet, He gas duct, and aperture. - The difference between scanning with multipurpose and PBS dedicated nozzle is the existence of other nozzle components, especially the MLC and He gas pipe.

**Fig 1:**
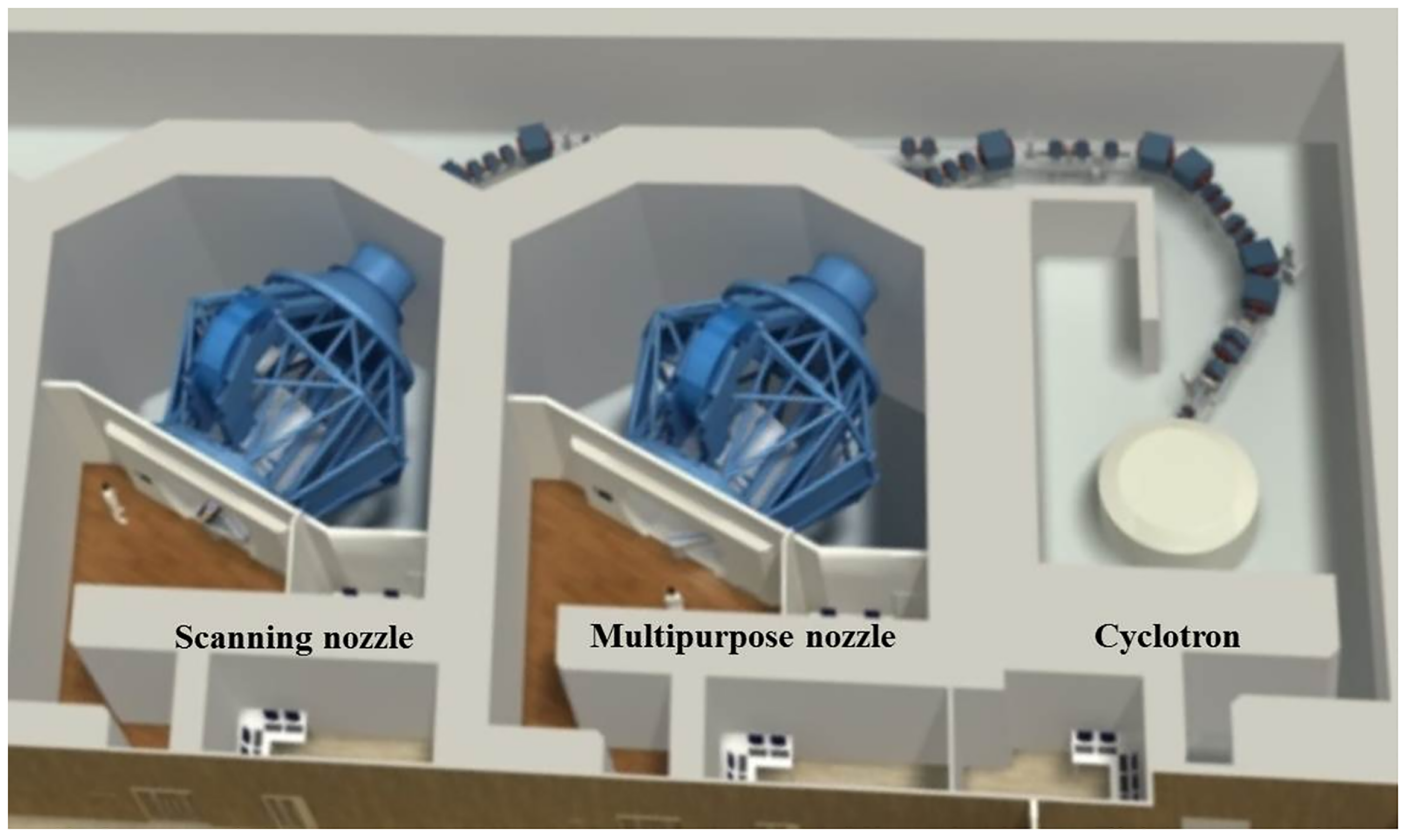
Schematic of SMC proton therapy facility.

**Fig 2:**
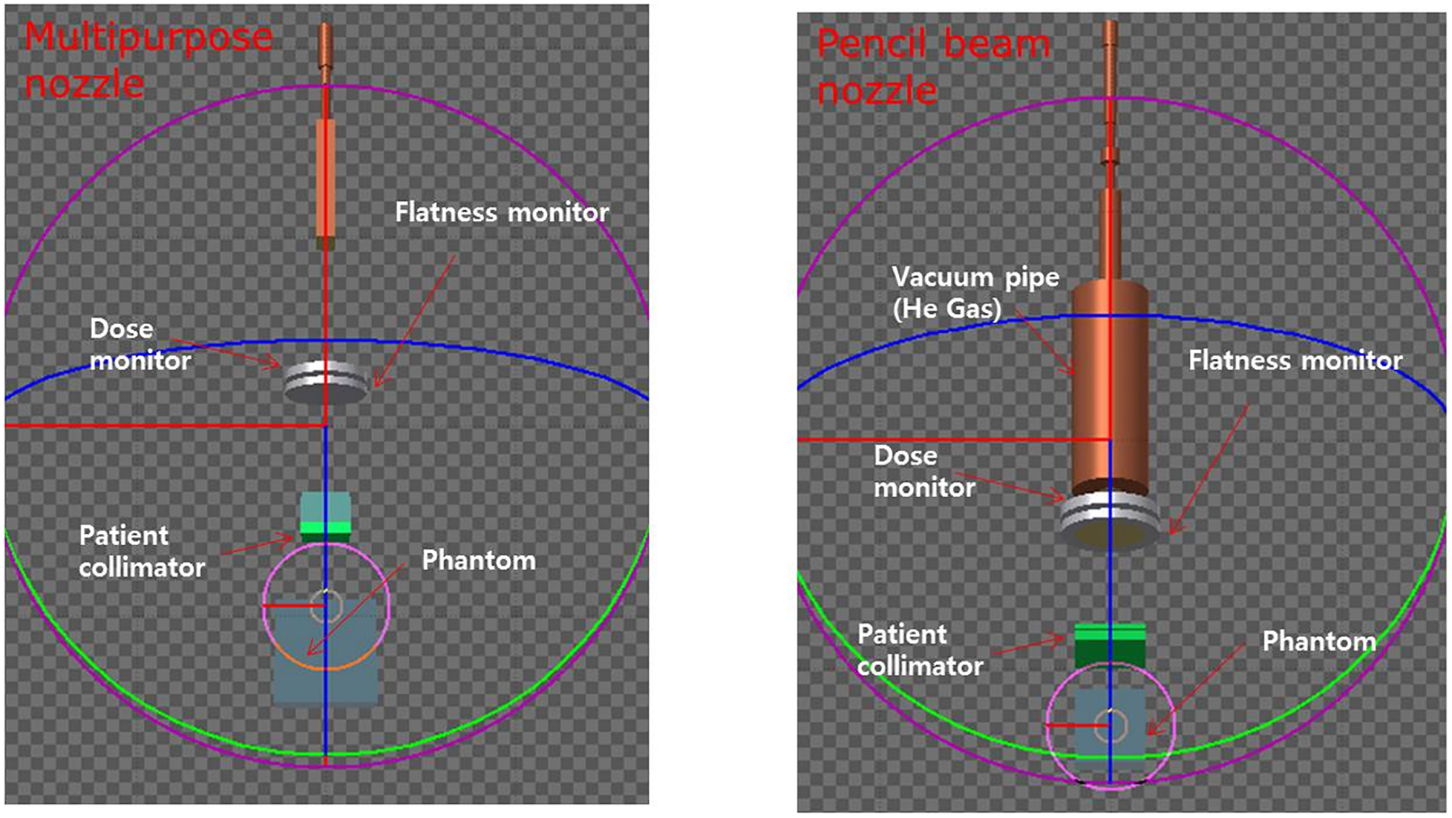
Design of each scanning nozzle using Monte Carlo simulation FLUKA.

Fig 1 shows a schematic of the proton therapy facilities at SMC and Fig 2 indicates the scanning mode nozzles that were modeled using FLUKA simulation to verify the beam properties. For both types of nozzle, FLUKA simulation was carried out. However, the experimental measurement was only performed at Gantry 1. To validate the proton therapy scanning nozzles of Gantries 1 and 2 in the scanning mode at SMC, we performed modeling using FLUKA Monte Carlo simulation code. Monte Carlo studies were performed using geometry information of the two different nozzles provided by the nozzle manufacturer.

**Fig 3:**
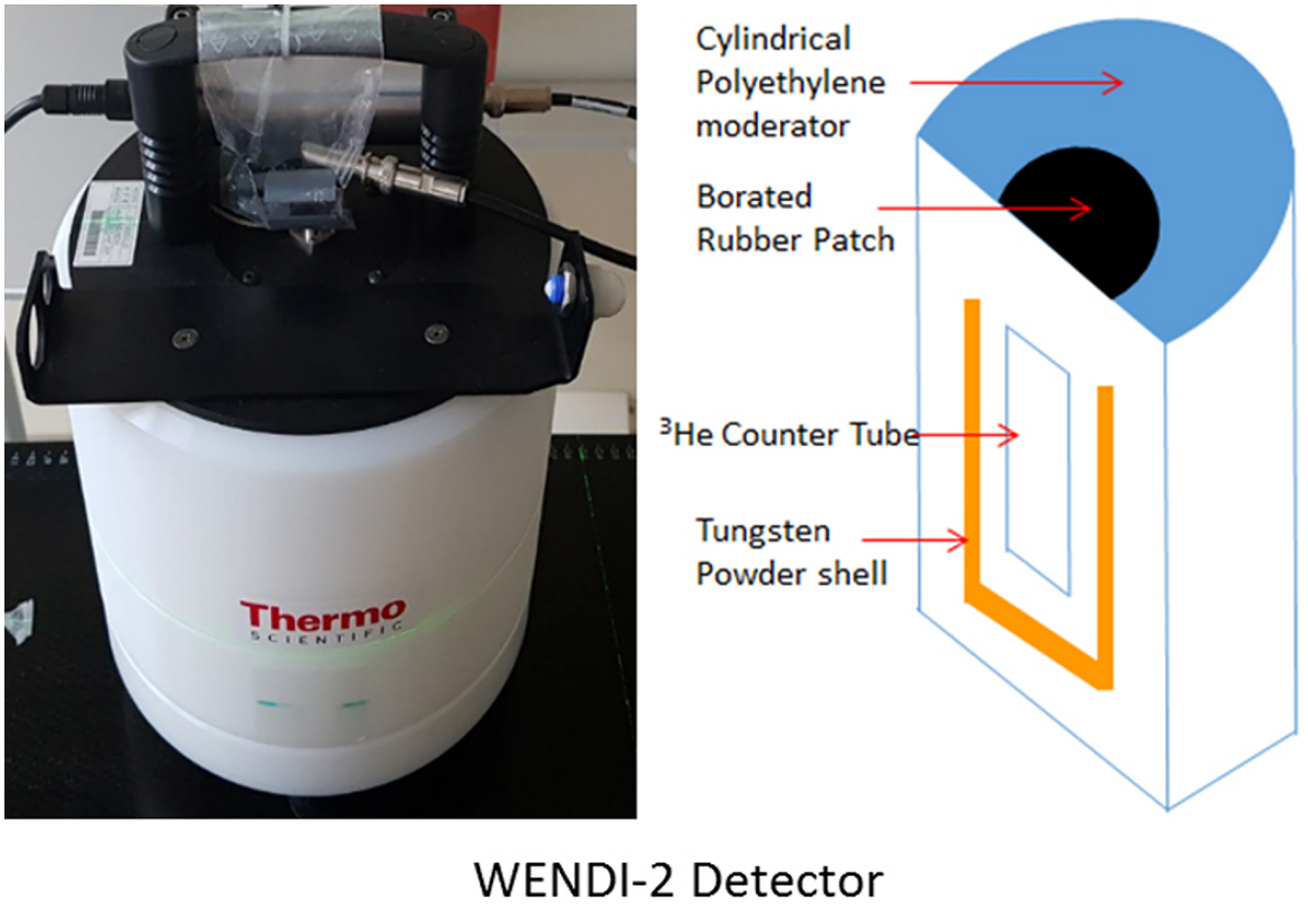
Feature of the WENDI-2 detector and its Schematic modeled in Monte Carlo simulation. (Left) The WENDI-2 neutron detector used for measurement of secondary neutron flux (Thermo Scientific), (Right) Schematic of the WENDI-2 detector structure that modeled in FLUKA simulation.

**Fig 4:**
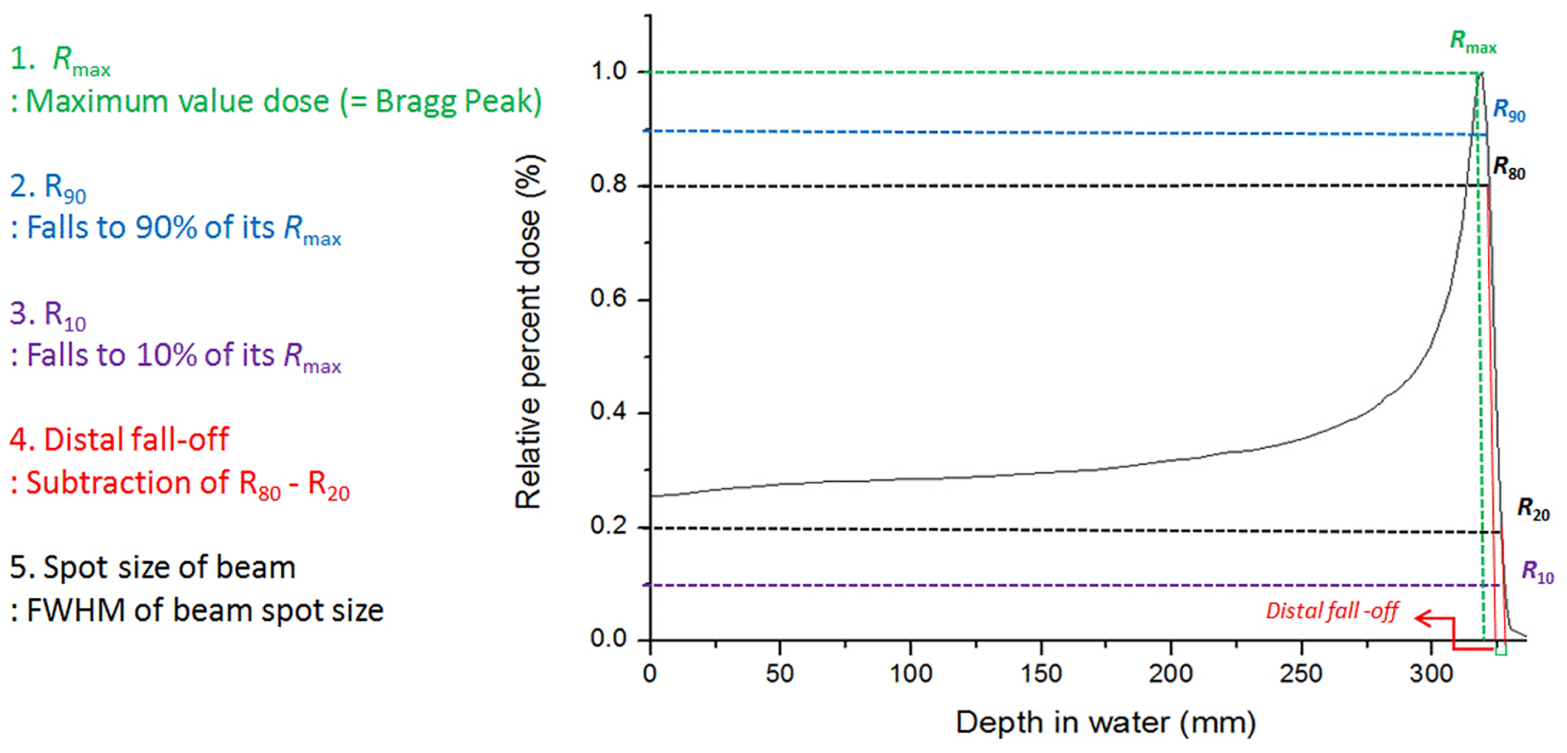
Parameters for data analysis of beam properties.

In this study, the neutron detector was modeling by WENDI-2 detector (Wide Energy Neutron Detection Instrument, Thermo Scientific, USA). Fig 3 depicts WENDI-2 detector. To validate simulation system, the Monte Carlo simulation was performed evaluating proton beam properties such as the Bragg peak, distal fall-off, R_90_, R_10_, and spot size of the beam based on experimental beam data. Fig 4 is explained used beam parameters for the proton beam properties. A 40 × 40 × 40 cm of plastic water phantom was placed around the gantry radially from 0° to 270° with 30 different beam energies from 70 MeV to 230 MeV.

**Fig 5:**
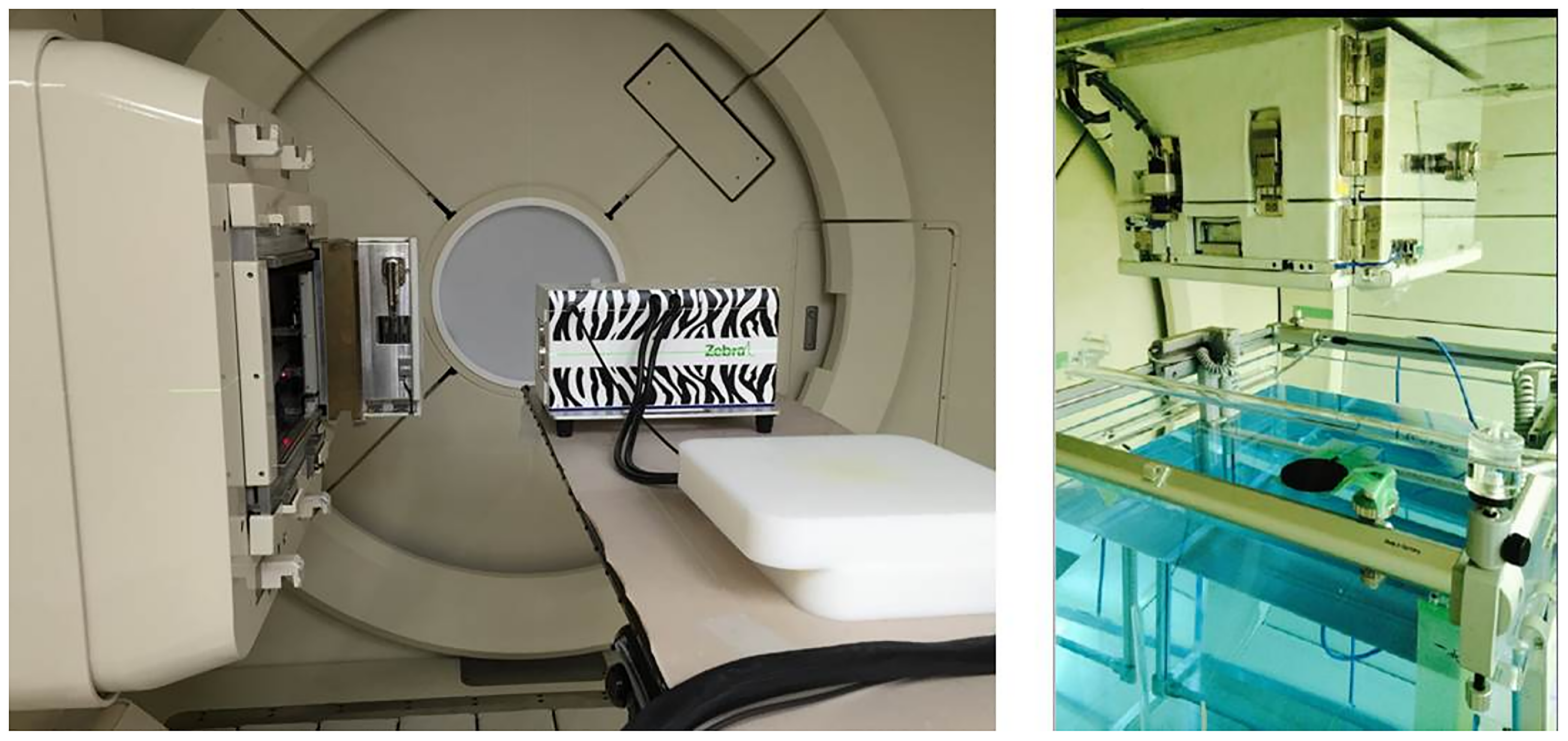
Experiment equipment to obtain proton beam properties. (Left) Installation of the ion chamber on the patient table to measure the Bragg peak, R_90_, R_10_ and distal fall-off (Zebra multi-layer ion chamber, IBA Inc.), (Right) Installation of the ion chamber in the water phantom for beam spot size measurement.

To obtain experimental results, we are performed measurement using Zebra (IBA), water phantom and ion chamber in same simulation conditions. Fig 5 shows the experiment figure to obtain proton beam properties.

### 2.2 Measurement of secondary neutrons generated by each nozzle

The secondary neutrons are produced by the nuclear interactions with the material in the beam path during treatment. This issue remains an unavoidable problem of any proton therapy technique including wobbling, scattering, and scanning. Although secondary neutron exposure is relatively low to compare with the therapeutic protons, the secondary neutrons provide a significant contribution to the integral dose and may cause secondary cancer [15–16].

There are two sources of secondary neutron in proton therapy: external neutrons from other materials and internal neutrons generated inside the patients. The external neutrons are dependent on the design of the proton therapy nozzle and treatment devices within the beam line. On the other hand, the internal neutrons are dependent on the beam range and field size in the patient. In the secondary neutron measurement, sum of the two sources are shown as the ambient neutron dose at any adjacent positions. Therefore, only external neutron dose can be measured when phantom is not installed on the beam line. In other words, the influence of the internal neutron dose can be derived when phantom is located after the external neutron dose has been measured.

In this study, we measured ambient neutron dose during the scanning mode proton therapy with Gantry 1 using a WENDI-2 neutron detector in both simulation and experiment. In this simulation, the wall of therapy room was not considered and the effect was neglected. To determine the dose contribution from only the external neutrons, the secondary neutron dose was measured at 4 positions (x-axis off set -44.29 cm, -16.43 cm, 16.43 cm and 44.29 cm) without the phantom. An irradiation of 110 MeV of proton energy was produced by Gantry 1.

**Fig 6:**
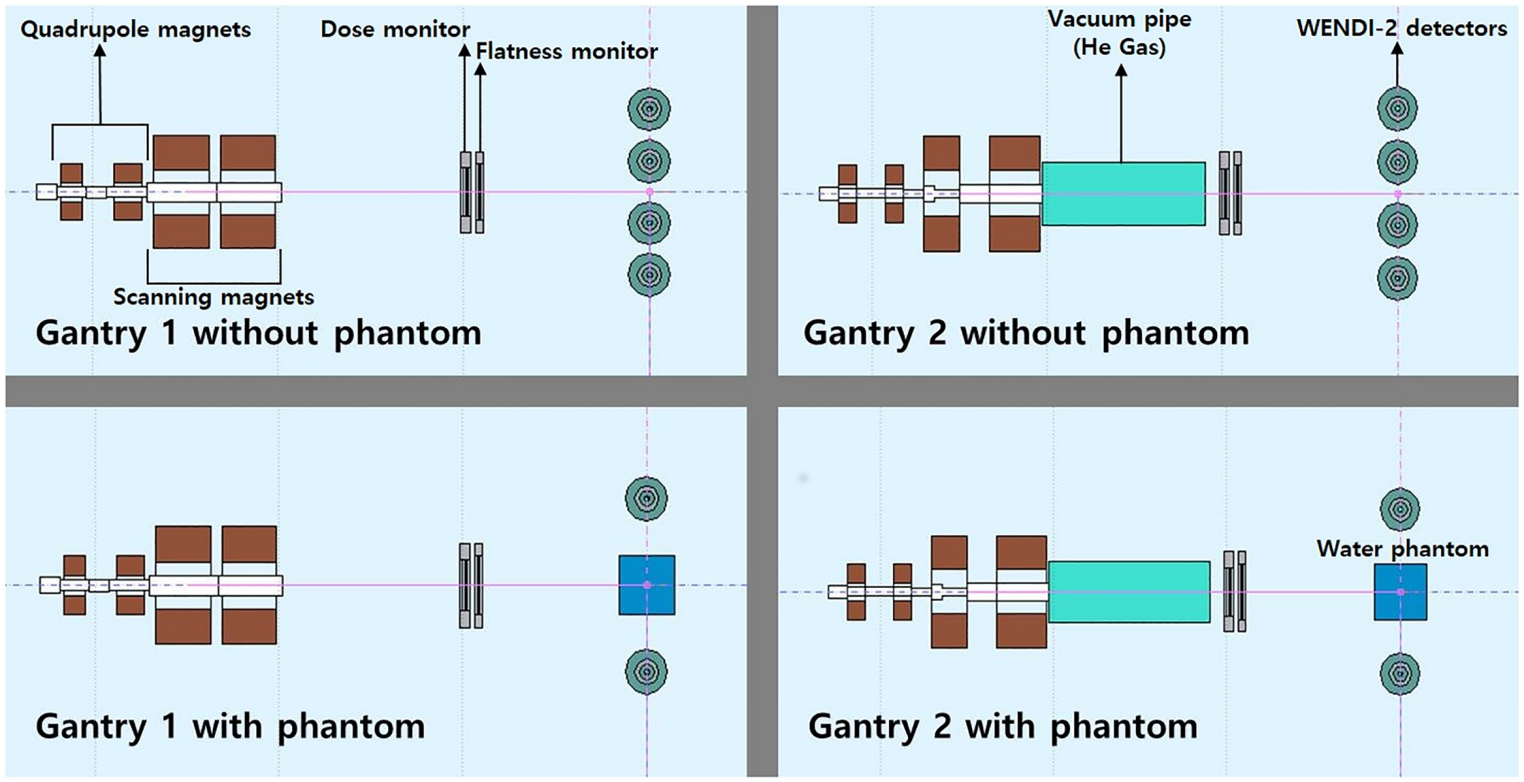
Measurement of secondary neutrons in each gantry by FLUKA.

**Fig 7:**
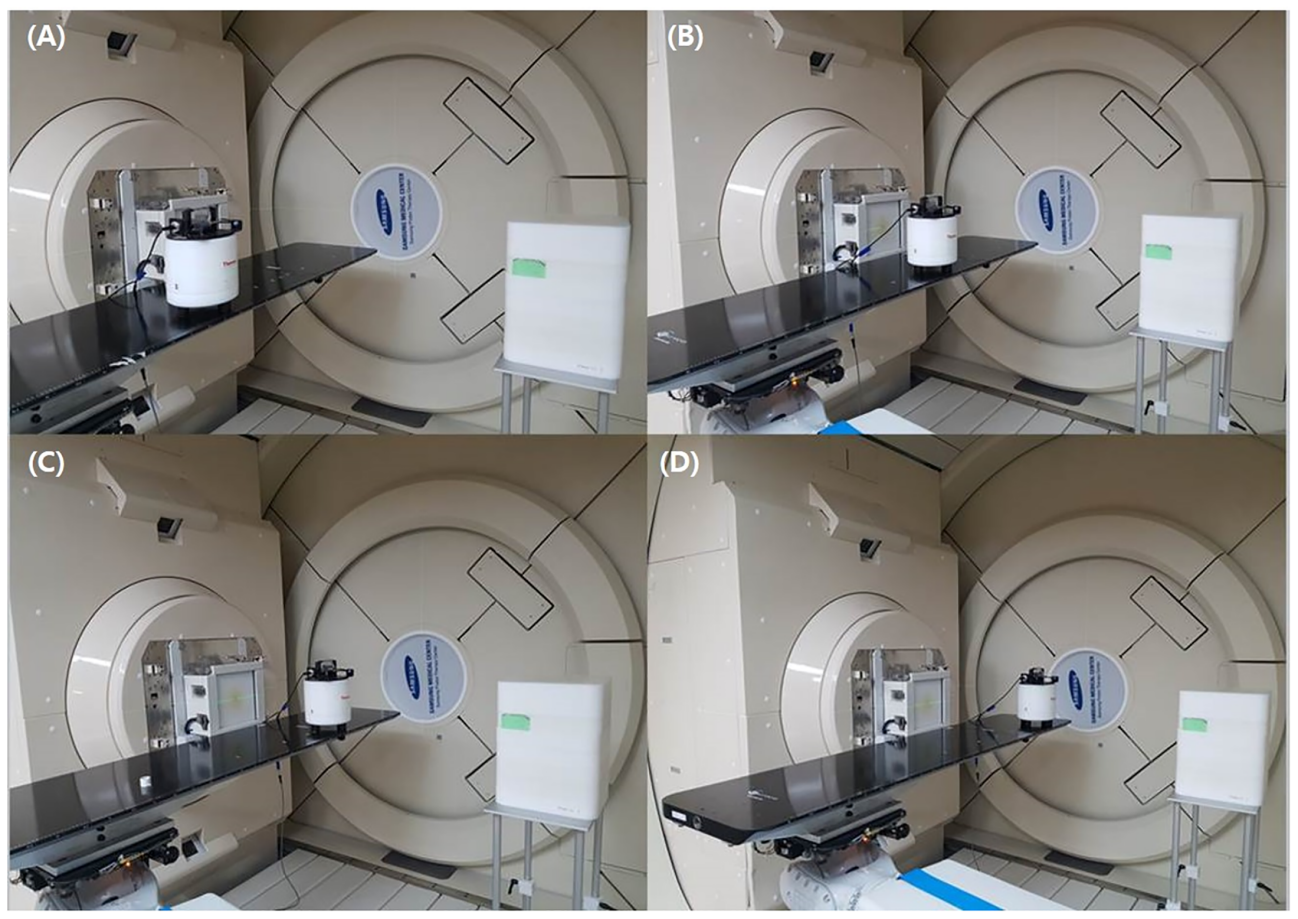
WENDI-2 detector location for secondary neutron dose measurement. x-axis offset of (A) 44.29 cm, (B) 16.43 cm, (C) -16.43 cm and (D) -44.29 cm, respectively.

After the external neutron has been evaluated by the ambient neutron dose, a 30 × 30 × 30 cm of plastic water phantom was positioned at the isocenter to investigate the influence of the internal neutron dose. Fig 6 shows the simulation design of Gantries 1 and 2. In the experiment, the measurement was performed at Gantry 1 with an identical condition of the simulation. Fig 7 shows the experimental setup for measurement of the external secondary neutron from the scanning nozzle.

## 3. Results and discussion

### 3.1 Proton scanning validation using Monte Carlo simulation

**Fig 8:**
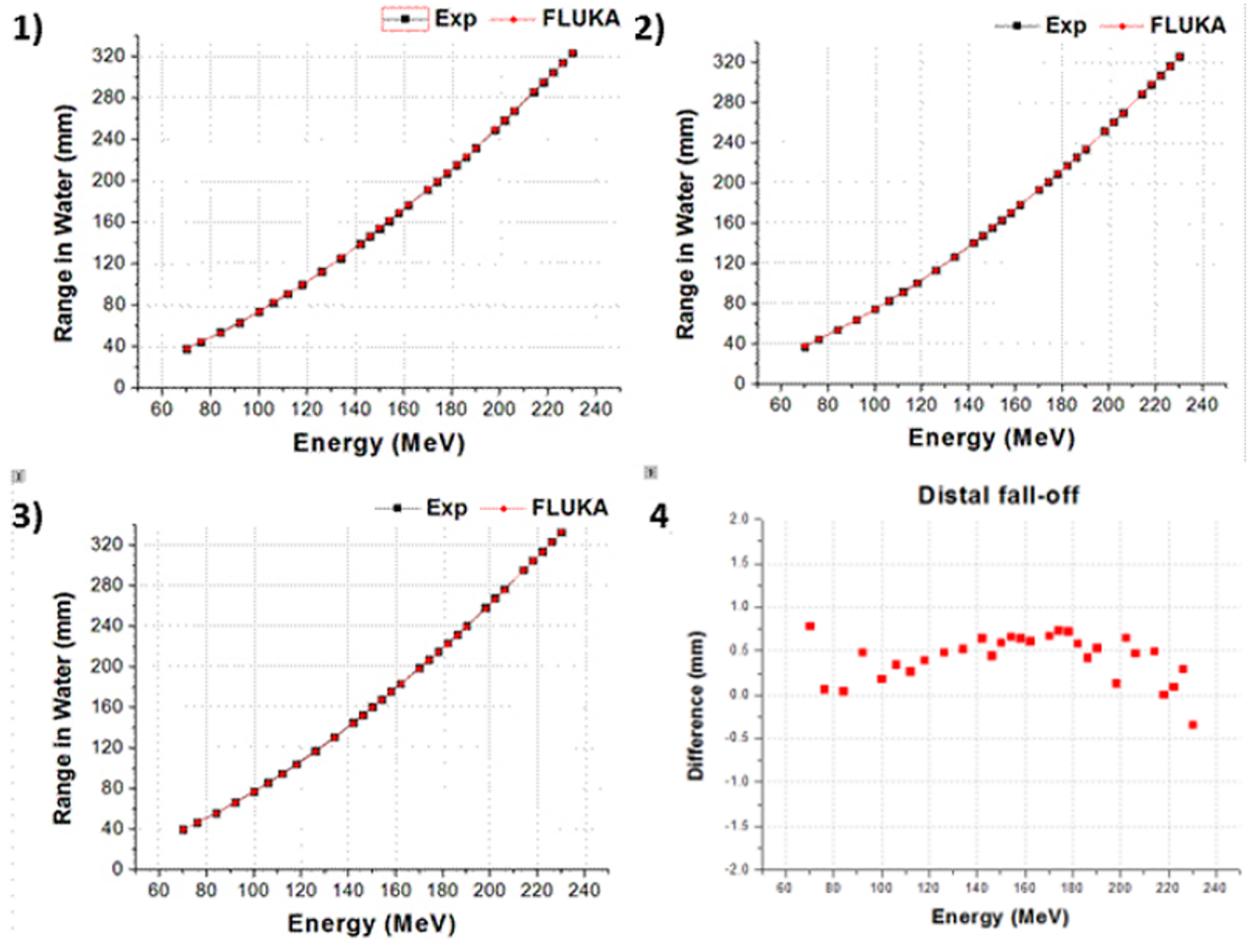
Gantry 1 results: 1) Bragg peak, 2) R90, 3) R10, and 4) distal fall-off.

**Fig 9:**
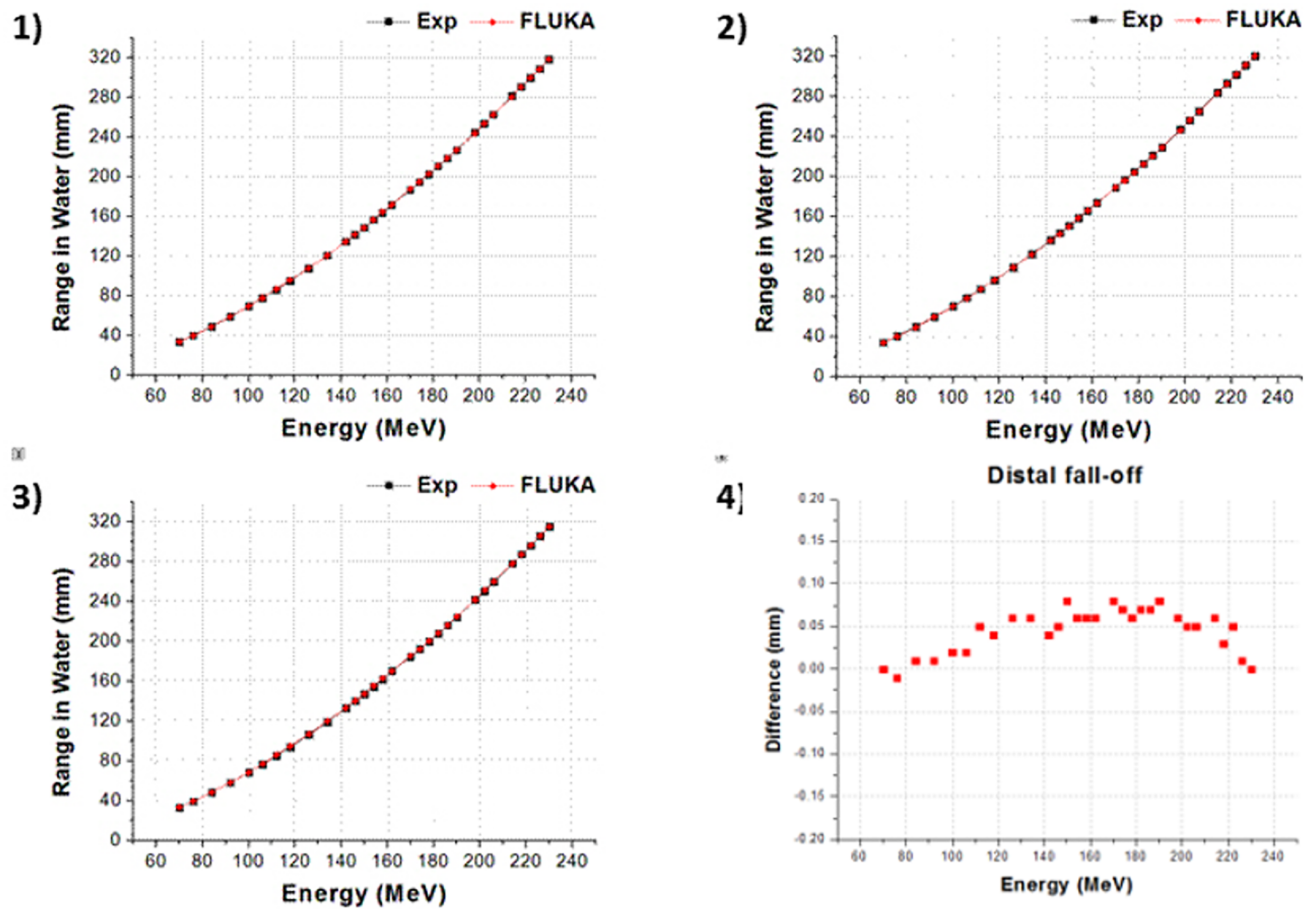
Gantry 2 results: 1) Bragg peak, 2) R90, 3) R10, and 4) distal fall-off.

**Fig 10:**
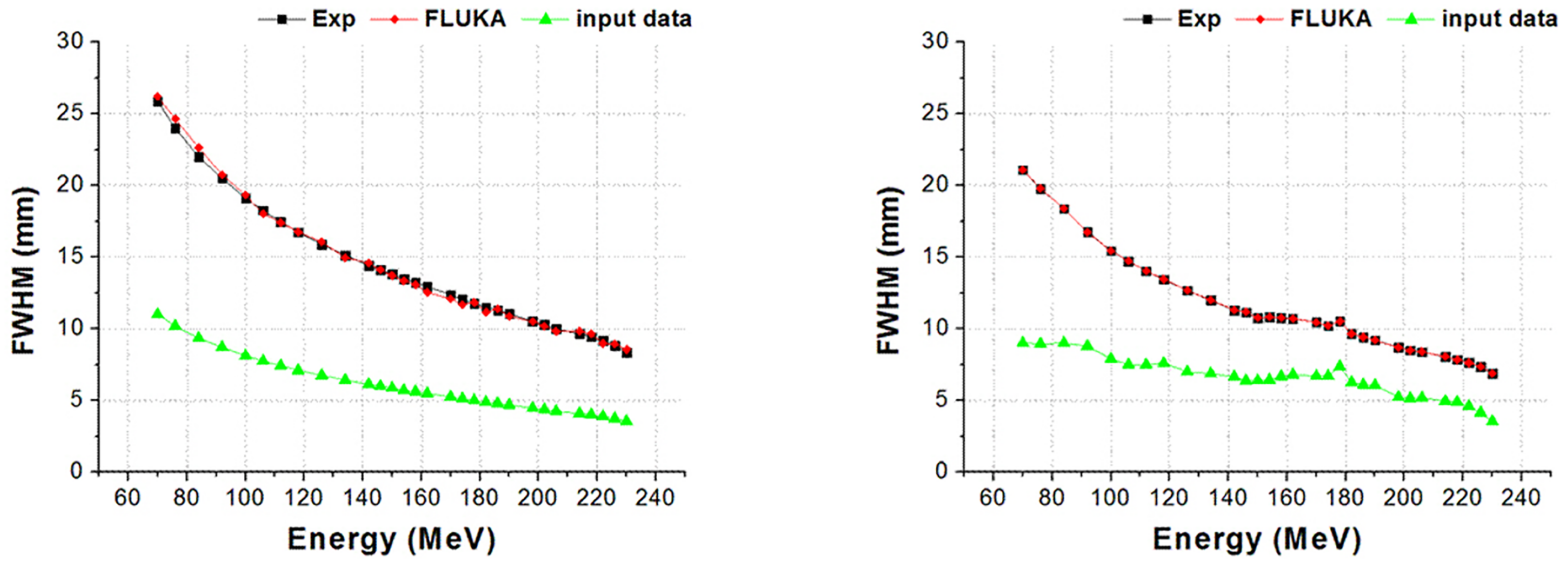
Results from Gantries 1(Left) and 2(Right) of FWHM of beam spot size.

We designed and simulated the two different proton therapy nozzles at SMC. Figs 8 and 9 show the difference of beam properties between the simulation data and experimental data at Gantries 1 and 2, respectively. Fig 8 shows the result of beam properties that compared the experimental data and simulation data at gantry 1 scanning nozzle. Also, Fig 9 shows the result at scanning nozzle of gantry 2. The difference between the experimental data and Monte Carlo beam validation results of Bragg peak, R_90_, R_10_, and distal fall-off are less than 1 mm. Additionally, Fig 10 indicates that the difference of beam spot size in terms of the FWHM is less than 0.5 mm at both Gantries 1 and 2. In the difference comparison, the simulation result show an agreement with the experimental data within 2.5% of error range.

### 3.2 Measurement of the secondary neutron from each nozzle

**Fig 11:**
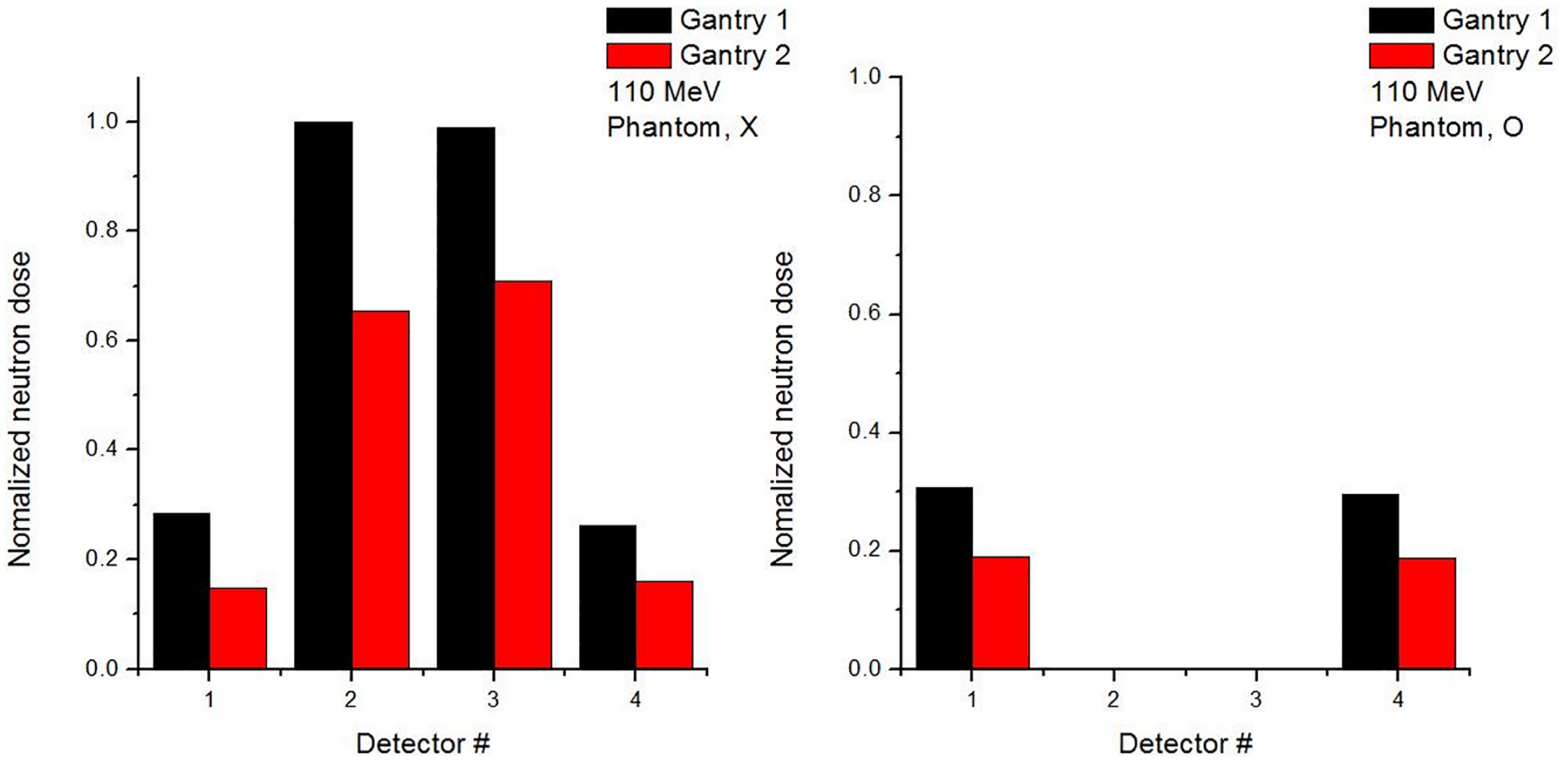
Normalized secondary neutron dose from 110 MeV proton beam at Gantries 1 and 2 from Monte Carlo simulation.

**Fig 12:**
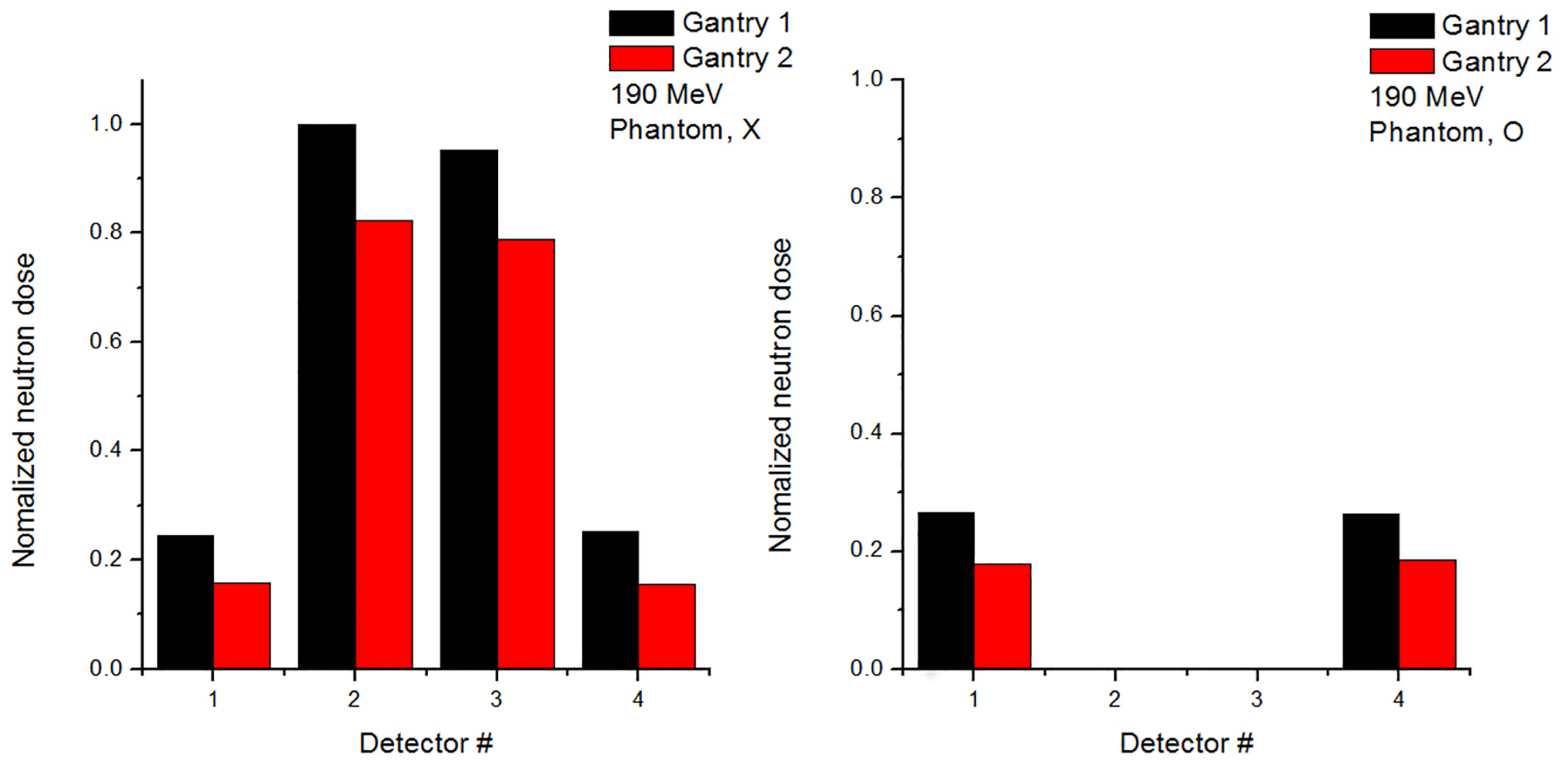
Normalized secondary neutron dose from 190 MeV proton beam at Gantries 1 and 2 from Monte Carlo simulation.

To distinguish between internal and external neutrons, Monte Carlo code was used to simulate the delivery of the proton beam with and without a phantom. The neutron detector was simulated as a WENDI-2. Figs 11 and 12 show simulation results of the detected value of ambient neutron dose with beam energies of 110 and 190 MeV, respectively. Also, Figs 11 and 12 indicate ambient neutron doses normalized to the maximum value at the gantry 1 and 2, respectively.

The normalized neutron flux at Gantry 1 is higher than the flux of Gantry 2, and the results at positions 1 and 4 show similar tendencies because detectors are located symmetrically from the isocenter, as do those at positions 2 and 3. For positions 1 and 4, the neutron dose with phantom is higher than the dose without phantom because internal neutrons from the phantom are produced in addition to the external neutrons.

### 3.3 Comparison between Monte Carlo simulations and experimental measurements

**Fig 13:**
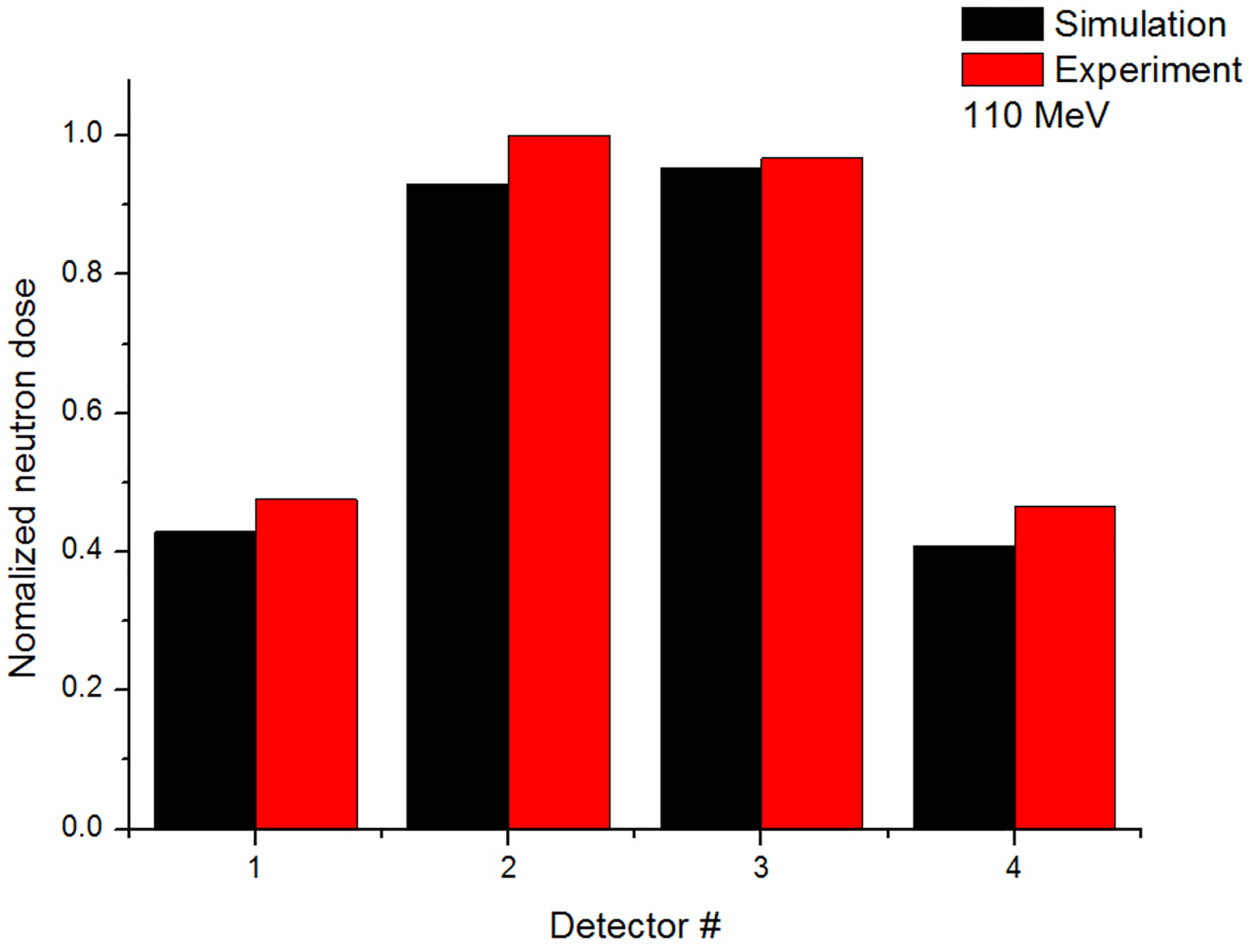
Comparison of simulated and experimentally measured values of normalized.

To compare the simulated and experimentally measured ambient neutrons dose at Gantry 1 in the same conditions, the secondary neutrons ambient dose was measured using the WENDI-2 detector. The secondary neutron dose was measured at 4 positions without a phantom with a 110 MeV proton beam. Fig 13 shows the comparison of the ambient neutron dose acquired from the simulation and experiment. The results were normalized to the maximum value of the experiment. The experimental and simulation values show similar tendency that the number of external neutrons reduces as the detection distance from the isocenter is increased. Also, the results at positions 1 and 4 show similar tendencies because detectors are located symmetrically from the isocenter, as do those at positions 2 and 3. These results prove that the simulation data provides reliable data of the induced neutron dose

## 4. Conclusions

In this study, we evaluated secondary neutron production from the proton beam-scanning nozzle. In order to assess the secondary neutron production, we modeled the proton scanning nozzle system using FLUKA Monte Carlo simulation and verified its validity. The verification process was that comparison beam properties achieved from the simulation and experiment in terms of the Bragg peak, R_90_, R_10_, distal fall-off and FWHM of the beam spot size. After the FLUKA modeling was confirmed, the secondary neutron production was measured by the secondary neutron dose from the simulation and experiment, respectively. As results of the beam properties comparison, the proton scanning nozzle modeling using FLUKA simulation was verified that describes the actual scanning nozzle with the high precision. Using the validated nozzle modeling, the amount of secondary neutron production was estimated and compared to the experimental result. The result can be utilized to the further study which considers the shielding material design for secondary neutron reduction. In other words, the amount of secondary neutron production which is reduced depending on the shielding material design can be predicted by using the FLUKA simulation modeling. In addition, these results will contribute to provide the knowledge for the development of radiation safety technology in medical particle accelerators.

## References

[1] Miller DW, A review of proton beam radiation therapy, Med. phys. (1995) 22 doi: 10.1118/1.597435 8587548 10.1118/1.597435

[2] Olsen DR, Bruland OS, Frykholm G, Norderhaug IN. Proton therapy—a systematic review of clinical effectiveness. Radiother Oncol. (2007) 83(2):123–32. http://dx.doi.org/10.1016/j.radonc.2007.03.001 17499374 10.1016/j.radonc.2007.03.001

[3] Shin JS, Shin E, Han Y, Ju SG, Kim JS, Ahn SH, et al Analysis of changes in dose distribution due to respiration during IMRT. Radiat Oncol J. 2011;29(3):206–13. doi: 10.3857/roj.2011.29.3.206 22984672 10.3857/roj.2011.29.3.206 PMC3429904

[4] Lawrence TD, NCRP Report, No. 171. uncertainties in the estimation of radiation risks and probability of disease causation, Med. phys. (2014) 10.1118/1.4869175

[5] Newhauser WD, Zhang R, The physics of proton therapy. Phys Med Biol(2015). doi: 10.1088/0031-9155/60/8/R155 25803097 10.1088/0031-9155/60/8/R155 PMC4407514

[6] Schneider U, Lomax A, Lombriser N. Comparative risk assessment of secondary cancer incidence after treatment of Hodgkin's disease with photon and proton radiation. Radiat Res 2000;154(4):382–388. 11023601 10.1667/0033-7587(2000)154[0382:craosc]2.0.co;2

[7] Paganetti H. Nuclear interactions in proton therapy: Dose and relative biological effect distributions originating from primary and secondary particles. Phys. Med. Biol. 2002, 47, 747–764. https://doi.org/10.1088/0031-9155/47/5/305 11931469 10.1088/0031-9155/47/5/305

[8] Zacharatou JC, Paganetti H, Risk of developing second cancer from neutron dose in proton therapy as function of field characteristics: organ and patient age. Int. J. Radiat. Oncol. Biol. Phys. 82 228–35 (2008), https://doi.org/10.1016/j.ijrobp.2008.04.069 10.1016/j.ijrobp.2008.04.069 18571337

[9] Kim JS, Shin JS, Kim DH, Shin EH, Chung KZ, Cho SK, et al, Feasibility Study of Neutron Dose For Real Time Image Guided Proton Therapy: A Monte Carlo Study. JKPS. 2015; Volume 67, issue 1, pp 142–146. https://doi.org/10.3938/jkps.67.142

[10] Yonai S, Matsufuji N, Kanai T, Matsui Y, MAtsushita K, Yamashita H et al, Measurement of neutron ambient dose equivalent in passive carbon-ion and proton radiotherapies. Med Phys, 35(2008) pp 4782–4792. https://doi.org/10.1118/1.2989019 19070210 10.1118/1.2989019

[11] Farah J, Mares V, Romero-Exposito M, Trinkl S, Domingo C, Dufek V. et al, Measurement of stray radiation within a scanning proton therapy facility: EURADOS WG9 intercomparison exercise of active dosimetry systems. Med Phys. 42(5), 2572–2584 (2015) https://doi.org/10.1118/1.4916667 25979049 10.1118/1.4916667

[12] Hall E.J.., Intensity modulated radiation therapy, protons, and the risk of second cancers Int J Radiat Ooncol Biol Phys, 65 (2006), https://doi.org/10.1016/j.ijrobp.2006.01.027 10.1016/j.ijrobp.2006.01.027 16618572

[13] A. Ferrari, P. R. Sala. World Scientific. P. Dragovitsch, S. L. Linn, M. Burban, A new model for hadronic interactions at intermediate energies for the FLUKA code, Proc. of the MC93 International Conference on Monte Carlo Simulation in High Energy and Nuclear Physics, Tallahassee, Florida, 22–26 February 1993, p 277–288.

[14] Chung KZ, Han YY, Kim JS, Ahn SH, Ju SG, Jung SH et al, The first private-hospital based proton therapy center in Korea; Status of the Proton Therapy Center at Samsung Medical Center. Radiat OncoL J. 2015; 33(4):1–7 doi: 10.3857/roj.2015.33.4.337 26756034 10.3857/roj.2015.33.4.337 PMC4707217

[15] Inskip PD, Curtis RE. New malignancies following childhood cancer in the United States, 1973–2002. Int J Cancer 2007;121:2233–2240. https://doi.org/10.1002/ijc.22827 17557301 10.1002/ijc.22827

[16] Ning MS, Perkins SM, Dewees T, Shinohara ET,. Evidence of high mortality in long term survivors of childhood medulloblastoma. J Neurooncol 2015;122:321–327. https://doi.org/10.1007/s11060-014-1712-y 25557108 10.1007/s11060-014-1712-y

